# Response to the commentary by Xu et al. on the PV-AIM study

**DOI:** 10.1038/s41375-025-02805-1

**Published:** 2025-11-17

**Authors:** Florian H. Heidel, Martin Griesshammer, Jean-Jacques Kiladjian

**Affiliations:** 1 Hematology, Hemostasis, Oncology and Stem Cell Transplantation, Hannover Medical School (MHH), Hannover, Germany; 2 Leibniz Institute on Aging, Fritz-Lipmann-Institute, Jena, Germany; 3 University Clinic for Hematology, Oncology, Hemostaseology and Palliative Care, Johannes Wesling Medical Center Minden, UKRUB, University of Bochum, Bochum, Germany; 4 Universite ´ de Paris, AP-HP, Hôpital Saint-Louis, Centre d’Investigations Cliniques, INSERM, CIC1427, Paris, France; 5 Sezione di Ematologia, Dipartimento di Scienze Radiologiche ed Ematologiche, Università Cattolica, Fondazione Policlinico A. Gemelli IRCCS, Roma, Italy; 6 Praxis Interdisziplinäre Onkologie und Hämatologie, Freiburg, Germany; 7 Luebecker Onkologische Schwerpunktpraxis, Luebeck, Germany; 8 Novartis Pharma GmbH, Nuremberg, Germany; 9 Novartis Ireland Limited, Dublin, Ireland

**Keywords:** Myeloproliferative disease, Risk factors

We read with interest the commentary by Xu et al. [1], who highlight important conceptual aspects of resistance definitions, symptom burden, and confounding variables in HU-treated PV. These reflections underscore the need for comprehensive evaluation of both biological and clinical risk factors and emphasize the importance of patient-centered outcomes. However, several points in their letter warrant clarification. Most importantly, their critique does not fully account for the methodology, scope, and objectives of the PV-AIM study [2], nor does it recognize the specific strengths of an artificial intelligence (AI)-driven machine learning (ML) approach to large-scale real-world data.

## The unmet need in prognostic modeling for PV and MPNs

The management of myeloproliferative neoplasms (MPNs) continues to face a pressing unmet need: the lack of robust prognostic models that adequately capture the complexity of disease progression and treatment resistance. Despite decades of clinical and translational research, patients with MPNs—and in particular those with polycythemia vera (PV)—continue to experience considerable toxicity and morbidity. Hydroxyurea (HU), as the most widely used cytoreductive therapy, is both indispensable and imperfect. A substantial proportion of patients develop resistance or intolerance, yet clinicians remain without precise parameters to anticipate such outcomes early. As a result, patients often undergo prolonged ineffective treatment, accumulating toxicity and carrying an elevated risk of thromboembolic events and disease progression [3].

PV represents a particularly valuable model disease in this regard. Unlike other MPN subtypes, PV is characterized by near-universal presence of a JAK2-activating driver mutation [4] and well-established diagnostic criteria [5]. This relative homogeneity reduces biological noise and provides an exceptional platform for testing novel analytic approaches aimed at identifying early predictors of therapy failure. We therefore consider PV uniquely suited for advanced prognostic modeling and an appropriate setting to leverage machine learning–based strategies such as those applied in the PV-AIM study [2].

## Machine learning and the OPTUM database: benefits and limitations

The PV-AIM study is, by design, a machine learning analysis using the Optum® electronic health record (EHR) database [6]. As such, it differs fundamentally from controlled clinical trials or registry studies. The central strength of this methodology lies in its ability to screen vast numbers of patients across hundreds of variables without the constraints of pre-selection or investigator bias.

The Optum® EHR dataset is one of the largest and most comprehensive resources available, comprising records of >90,000 patients with PV and offering >350 clinical and laboratory parameters per patient. This scale is unprecedented in a rare hematologic malignancy and allows for unbiased identification of potential predictors of HU resistance. As noted in recent publications who first described the MPN cohort within Optum [6], the database provides an unparalleled opportunity to study disease patterns, treatment outcomes, and predictors of risk at population level.

We acknowledge, as the commentary authors rightly point out, that insurance and EHR databases have inherent limitations. Data capture is not protocol-driven, leading to missingness, variable granularity, and absence of structured symptom assessments. Nevertheless, the breadth of available information offers a complementary perspective to trial-based cohorts. While symptoms, quality-of-life measures, or certain comorbidities cannot be retrieved reliably from Optum, these factors are not ignored but rather reserved for the second step of validation in prospective studies. It is precisely this two-step process—first, unbiased signal generation in a large real-world dataset; second, hypothesis testing and refinement in controlled clinical trials—that defines the strength of the PV-AIM program.

Importantly, the commentary does not discuss the added value of machine learning itself. ML enables the simultaneous evaluation of interactions among hundreds of variables, identifying non-linear patterns and synergistic thresholds (e.g., the interplay of red cell distribution width [RDW] and hemoglobin [HGB]) that would be impossible to detect in conventional analyses. Such capabilities highlight the benefit of AI-guided modeling in rare diseases, where the complexity of biological and clinical predictors exceeds the capacity of traditional statistical approaches.

## Contextualizing confounders and symptom burden

The authors of the commentary argue, correctly, that comorbidities such as iron deficiency and patient-reported symptoms (e.g., splenomegaly, pruritus, or fatigue) are highly relevant to treatment response [7, 8]. These parameters, however, cannot be systematically extracted from an insurance database. Their absence is a limitation inherent to RWE analyses, not a flaw of the PV-AIM design. Importantly, this does not preclude validation of these factors in subsequent studies. Indeed, the PV-AIM program explicitly foresees integration of patient-reported outcomes (PROs) and symptom burden via the MPN-SAF in the prospective HU-F-AIM trial.

It should also be stressed that the PV-AIM publication in Leukemia does not claim to provide a definitive prognostic scoring system. Rather, it identifies easily measurable hematologic thresholds (RDW ≥ 17% and HGB ≤ 15.5 g/dL) as candidate predictors of HU resistance. These findings are not proposed to replace established clinical or molecular risk factors but to complement them and to stimulate further investigation.

## From hypothesis generation to validation: HU-F-AIM

Perhaps the most significant omission in the commentary is the lack of recognition of the HU-F-AIM trial. PV-AIM was conceived as a hypothesis-generating study. Its logical continuation is HU-F-AIM, a prospective, interventional, phase IV study currently underway. HU-F-AIM is designed precisely to address the limitations of retrospective, database-driven analyses by incorporating prospective data capture, HU dose escalation, molecular profiling, and PROs via MPN-SAF.

This stepwise approach ensures that the thresholds identified in PV-AIM are rigorously validated in a real-world, yet controlled, clinical setting. HU-F-AIM therefore provides the “granularity” called for in the commentary and will directly test whether the AI-derived predictors hold when integrated with clinical, molecular, and symptom-based assessments. Far from neglecting the complexity of PV, the PV-AIM/HU-F-AIM continuum is a stringent and rational model of translational research: broad signal detection in RWE, followed by prospective validation in targeted trials.

## Clinical utility versus algorithmic scoring

The commentary appears to misinterpret the intent of PV-AIM as offering a new prognostic algorithm. This is not the case. Rather, PV-AIM identifies simple, clinically accessible laboratory values that may serve as early “red flags” for HU resistance. The rationale is pragmatic: in routine practice, physicians can easily assess RDW and HGB without additional cost or effort. Recognizing patients with abnormal values as being at higher risk of HU failure allows for closer monitoring and earlier consideration of second-line therapy. In this way, PV-AIM findings are meant to complement—not supplant—existing prognostic models.

## The unique contribution of AI-guided analyses in PV

Ultimately, the value of PV-AIM lies in its demonstration that advanced ML can be applied successfully in a homogeneous disease like PV to generate clinically meaningful predictors. By leveraging the largest available real-world dataset, PV-AIM introduces a novel paradigm: using AI to screen for risk factors in thousands of patients and then validating them prospectively in a dedicated trial. This approach may serve as a blueprint for other MPN subtypes and hematologic malignancies, where patient heterogeneity and lack of large datasets have long hampered the development of robust prognostic models.

## Conclusion

We welcome the engagement of Xu et al. with the PV-AIM study and appreciate their emphasis on confounders, symptom burden, and guideline-based definitions. At the same time, we respectfully note that their commentary does not account for the methodological nature and goals of PV-AIM, nor does it recognize the added value of AI-guided machine learning in a rare disease setting. The Optum dataset, while limited in granularity, provides an unparalleled opportunity to detect novel predictors at scale. The prospective HU-F-AIM trial is already underway to validate these predictors alongside clinical, molecular, and patient-reported parameters, thus addressing the very concerns raised.

In our view, this two-step process—unbiased signal generation followed by structured prospective validation—offers a stringent and innovative path toward improved prognostic modeling in PV. It is our hope that by integrating AI-driven discovery with clinical trial validation, we can move closer to precision medicine for patients with PV, reducing unnecessary toxicity and improving outcomes.
